# Assessing the play and learning environments of children under two years in peri-urban Lima, Peru: a formative research study

**DOI:** 10.1186/s12889-020-10119-3

**Published:** 2021-01-09

**Authors:** Jessica D. Rothstein, Audrey J. Buckland, Kristin Gagnier, Mayra Ochoa, Aliya Allen-Valley, Belinda Jivapong, Lilia Z. Cabrera, Elli Leontsini, Kelly R. Fisher

**Affiliations:** 1 Department of International Health, Johns Hopkins Bloomberg School of Public Health, Baltimore, MD USA; 2 Science of Learning Institute, Johns Hopkins University, Baltimore, MD USA; 3 Asociación Benéfica Proyectos en Informática, Salud, Medicina, y Agricultura (PRISMA), Lima, Peru

**Keywords:** Early child development, Formative research, Mixed methods, Peru, Peri-urban, Learning environment

## Abstract

**Background:**

Home-based interventions have potential for improving early child development (ECD) in low-resource settings. The design of locally acceptable strategies requires an in-depth understanding of the household context. In this formative research study, we aimed to characterize the home play and learning environments of children 6–23 months of age from low-income households in peri-urban Lima, Peru.

**Methods:**

Drawing on the developmental niche framework, we used quantitative and qualitative methods to understand children’s physical and social settings, childcare practices, and caregiver perspectives. We conducted interviews, unstructured video-recorded observations, and spot-checks with 30 randomly selected caregiver-child dyads, 10 from each child age group of 6–11, 12–17, and 18–23 months of age, as well as key informant interviews with 12 daycare instructors. We analyzed the data for key trends and themes using Stata and ATLAS.ti and employed an adapted version of the Indicator of Parent-Child Interaction to evaluate the observations.

**Results:**

Children’s social settings were characterized by multi-generational homes and the presence of siblings and cousins as play partners. Access to books and complex hand-eye coordination toys (e.g., puzzles, building blocks) in the home was limited (30.0 and 40.0%, respectively). Caregivers generally demonstrated low or inconsistent levels of interaction with their children; they rarely communicated using descriptive language or introduced novel, stimulating activities during play. Reading and telling stories to children were uncommon, yet 93.3% of caregivers reported singing to children daily. On average, caregivers ascribed a high learning value to reading books and playing with electronic toys (rated 9.7 and 9.1 out of 10, respectively), and perceived playing with everyday objects in the home as less beneficial (rated 6.8/10). Daycare instructors reinforced the problems posed by limited caregiver-child interaction and supported the use of songs for promoting ECD.

**Conclusions:**

The features of the home learning environments highlighted here indicate several opportunities for intervention development to improve ECD. These include encouraging caregivers to communicate with children using full sentences and enhancing the use of everyday objects as toys. There is also great potential for leveraging song and music to encourage responsive caregiver-child interactions within the home setting.

**Supplementary Information:**

The online version contains supplementary material available at 10.1186/s12889-020-10119-3.

## Background

Children’s early learning experiences profoundly shape health and development throughout their lifespan. Critical periods of cognitive, motor, and socio-emotional development begin during the prenatal period and peak during the first 2 years of life [1, 2]. Worldwide, it is estimated that approximately 250 million or 43% of children under 5 years of age are at risk of not achieving their full developmental potential [3]. These children are at a disadvantage from the first day of school, hindering future intellectual development and educational attainment, which often results in limited opportunities for employment and upward mobility during adulthood [4, 5]. Thus, poor early child development (ECD) sets off a cascade of events that reinforce the economic, social, and health inequities experienced by those who are most vulnerable in early life.

During the first 2 years of life, responsive caregiver-child interactions, nurturance, and access to stimulating, age-appropriate activities are key to fostering ECD [6–9]. Opportunities for play, as mediated by the caregiver, are another critical component of healthy learning environments [10]. Knowledge gained through play forms the foundation for more advanced cognitive development, as these activities allow children to attempt novel tasks, problem-solve, cooperate, and develop social skills [11–15]. Object play, for example, contributes to the development of sensory and motor skills, while social play helps children experiment with their language system and cognitive skills [11, 16–19].

For children growing up in low-income contexts, experiences take place primarily in the home environment. These children are less likely to be exposed to stimulating and appropriate caregiver-child interaction within the home [20–22]. In developing countries, access to learning materials for young children varies significantly by socio-economic status [23]. According to UNICEF’s Multiple Indicator Cluster Surveys from more than 100 low- and middle-income countries (LMICs), the availability of children’s books in the homes of children under 5 years of age ranged from 29.0% for the lowest wealth quintile to 56.6% for the highest wealth quintile [3]. Globally, it is estimated that 69.1% of children under 5 years of age receive adequate home stimulation (defined as recent exposure to at least four out of six basic activities), with an average disparity of 21.7 percentage points between the lowest and highest wealth quintiles. This disparity is most pronounced in Latin American and the Caribbean at 28.5 percentage points [24].

There is growing evidence that home-based interventions, in which community health workers (CHWs) or other paraprofessionals make home visits to provide training and support to caregivers, may mitigate the negative impact of poverty on ECD [25–27]. These interventions provide opportunities for CHWs to model stimulating activities with the child and, in some cases, to provide toys or picture books [28–31]. Given the variety of socio-economic conditions and cultural environments in low-resource communities throughout the developing world, the design of locally feasible and acceptable home-based interventions requires a contextualized understanding of household and caregiving practices. Nevertheless, research on the home learning environments in such settings remains scarce [6, 9].

These research gaps point to the need to focus on the “child-in-context” in order to effectively understand and address household-level influences on children’s early life experiences [32]. Super and Harkness’ (1986) concept of the “developmental niche” describes these influences as three integrated and interacting subsystems: 1) physical and social settings, 2) childcare customs and practices, and 3) caregivers’ psychology [32, 33]. In this model, “physical and social settings” captures features of the physical spaces (e.g., toys and books) and types of people where the child lives; key people include not only caregivers but also siblings, who are often important influences on children’s social and interpersonal skills [34, 35]. “Childcare customs and practices” refer to the patterns of behavior that are commonly used and accepted when interacting with children at a given developmental stage (e.g., how to carry or talk to an infant). Finally, “caregivers’ psychology” covers the perspectives and values that assign meaning to different practices and therefore organize caregiving strategies (e.g., whether speaking to an infant encourages language socialization). These three systems of the developmental niche are, in turn, influenced by broader macro-level factors such as poverty, employment, and food insecurity. This theory has guided research into a number of child-related topics, including eating practices among preschoolers in the U.S. and behavioral problems among children in Nepal [36, 37]. The developmental niche also provides a useful framework for examining the environmental factors shaping ECD during the first 2 years of life.

### Study objectives

In this exploratory study, we drew on the developmental niche framework to characterize the home learning environments of young children in peri-urban Lima, Peru, with an emphasis on play and communication. Focusing on the “child-in-context,” we used quantitative and qualitative methods to explore children’s physical and social settings, childcare practices, and caregivers’ perspectives as they relate to opportunities for play and caregiver-child interaction. In addition to caregiver and child participants, we also recruited local daycare instructors from the government-sponsored *Cuna Más* program to gain additional perspectives from individuals with intimate knowledge of ECD in this setting. The study comprised the first stage of formative research to develop an ECD intervention targeting caregiving behaviors in this population, and thus was used to identify needs, assets, and opportunities for improvement.

## Methods

### Study setting

The study was conducted in the shantytown district of *Villa El Salvador,* located on the southern outskirts of metropolitan Lima, Peru. Despite the country’s economic growth and improvements in many child health indicators in recent years, ECD remains a major challenge [38, 39]. Large disparities among wealth gradients have been documented for developmental measures among children under 2 years, as well as for language scores among 4- to 6-year-olds, and academic performance during secondary school [23, 40, 41]. The Peruvian government began investing in ECD in 1993 with the creation of the *Wawa Wasi* (‘Children’s Homes’ in Quechua) program, which provided public daycare services emphasizing safety, learning, nutrition, and health. While several studies have demonstrated that this program was highly valued by communities, evaluations of the effects on child growth and development have yielded mixed and inconclusive results [42]. The home environment of Peruvian children during their most sensitive developmental periods has not been well researched to date: according to UNICEF’s country profiles for the Nurturing Care Framework, which describes the essential components of successful ECD, data from Peru are entirely missing for the adequacy of responsive caregiving, early stimulation at home, and presence of books and playthings in the home [43].

The district of *Villa El Salvador* is a self-organized community that emerged over the past forty years as families migrated from the rural Andean highlands, built houses in informal settlements, and gradually established the zone’s urban organization and infrastructure [44, 45]. Currently, *Villa El Salvador* has roughly 393,000 residents; some live in well-established zones where families have full property rights, while others occupy makeshift settlements in recently developed squatter communities [46]. The primary language of the current generation of parents is Spanish and most residents self-identify as *mestizo* (of mixed ancestry); some elder residents speak Quechua either with or without Spanish. The communities of *Villa El Salvador* have historically engaged in co-management of public sector social programs such as *Wawa Wasi* and an in-kind food transfer program called *Vaso de Leche* (‘Glass of Milk’) [47]. The challenges of income and food insecurity remain common [48].

### Participants and sampling

Primary study participants were caregivers of children 6–23 months of age, as well as the children themselves. “Caregiver” was defined as the child’s parent or any other adult family member that regularly devotes substantial time (> 4 h/day) to childcare. Given that children undergo a range of developmental stages during the first 2 years of life, we aimed to collect data from three child age group tertiles (6–11 months, 12–17 months, and 18–23 months). We anticipated that approximately 10 caregiver-child dyads from each age group would allow us to reach saturation on key themes related to our research objectives [49]. This sample size was deemed sufficient given that our study population exhibited minimal ethnic and linguistic diversity. Caregiver-child dyads were not eligible for participation if the child was attending daycare, since we were focusing on the child’s home environment.

Field workers conducted door-to-door home visits in the four sectors comprising the field site to identify all eligible households and generate study interest. This resulted in a roster of all eligible and interested caregivers (*n*=134) out of approximately 900 households visited. We stratified these by child age group and randomly selected 10 caregivers from each group. To recruit participants, field workers made another home visit to describe the study procedures, with up to two follow-up attempts if a selected participant was initially unavailable. In cases where a selected participant was not eligible (due to enrolling in daycare or reaching 2 years of age in the time elapsed since the first home visit) or not reachable, she was replaced by a randomly selected replacement.

Other study participants were instructors at government-sponsored daycare centers of the *Cuna Más* program in the study site [50]. *Cuna Más* (‘More than a Crib’), run by Peru’s Ministry of Development and Social Inclusion (*Ministerio de Desarrollo y Inclusión Social,* MIDIS), was established in 2012 on the basis of the former *Wawa Wasi* program [42, 47]. Currently, *Cuna Más* supports daycare services in low- income urban areas as well as a home visiting service in rural areas. In our study context, daycare instructors—known locally as *madres-cuidadoras* (‘mother-carers’)—were eligible if they were at least 18 years of age and had been employed at a *Cuna Más* daycare center in the study site for at least 6 months. After receiving formal authorizations from the National *Cuna Más* Program and the Lima Metropolitan territorial office, we contacted all eligible instructors at the nine daycare centers in the field site.

### Data collection activities and measures

We collected data cross-sectionally between June and November 2018. Semi-structured and structured interviews with caregivers, spot-checks, and direct unstructured observations were conducted by two trained field workers who lived in neighboring communities and had substantial experience working with our target population. Two members of the study team trained in qualitative methods conducted key informant interviews with daycare instructors; this allowed one person to facilitate the interview while the other served as note-taker.

Members of the multidisciplinary study team developed all data collection instruments collaboratively and refined them with field worker input after pre-testing in the field site. This ensured that all questions were conveyed in a culturally appropriate manner and were compatible with the local vernacular. All data collection activities were conducted in colloquial Spanish in participants’ homes, or for daycare instructors, at the centers where they worked. The data collection methods are summarized in Table 1 and described in detail below.

**Table 1:** Data collection methods, goals, participants, and instruments

Method	Goal	Participants	Instrument
Semi-structured interview	Understand practices and perceptions around caregiving and healthy child development	30 caregiver-child dyads	Interview guide with open-ended questions and probes
Structured interview			Questionnaire with close-ended questions; adapted in part from Fisher et al. (2008)
Spot-check	Assess toys and learning materials available in the physical home setting		Standardized checklist adapted from “Learning materials” domain of HOME-IT
Unstructured observations	Assess the quality of caregiver-child interaction surrounding play and communication		30-min video-recorded observations rated according to adapted IPCI
Key informant interviews	Gather supplemental information on perceived barriers to ECD and learning within the home	12 daycare instructors	Interview guide with open-ended questions and probes

#### Semi-structured and structured interviews

We conducted interviews with caregivers (*N*=30) using an interview guide that was developed for this study and included both open-ended and close-ended sections (Supplement 1). Questions focused on exploring the child’s daily activities, whether and how the caregiver plays and interacts with the child, and caregiver perceptions of the relationships among play, learning, and healthy development.

For one section of the interview, we adapted structured procedures used by Fisher and colleagues (2008) to assess children’s engagement in play-based activities as well as caregivers’ perceptions of those activities [51]. Caregivers were asked how frequently the child engages in a series of activities, ranging from “never” to “every day.” Activities included those that required structured interaction or guidance from caregivers (e.g., having a story told to them) and those that did not (e.g., using building blocks, playing with electronic toys). We adapted items from Fisher et al.’s original tool to guarantee local relevance [51]. In addition, we asked caregivers to rate each activity in terms of its learning value, ranging from 1 (“This activity definitely does *not* set a foundation for learning”) to 10 (“This activity definitely sets a foundation for learning”).

Select socio-demographic characteristics including household size, water and sanitation infrastructure, and caregiver’s educational attainment were also collected. The interviews typically lasted between 40 and 75 min. Field workers audio-recorded all interviews with participant consent and documented responses through notes on structured paper forms.

#### Spot-checks

Spot-checks (*N*=30) were conducted in participants’ homes directly following the interviews to assess the presence of age-appropriate toys and other stimulating equipment. Field workers recorded the presence, quantity, and condition of both store-bought toys (e.g., blocks, figurines) and improvised or home-made toys (e.g., pot and spoon for banging) through a checklist on a standardized paper form. We drew the categories and types of toys from the “Learning materials” domain of the Home Observation for Measurement of the Environment – Infant/Toddlers Version (HOME-IT) scale, a widely used and validated measure of stimulation in the home [52–54].

#### Unstructured observations

We conducted thirty-minute direct unstructured observations (*N*=29) to assess the quality and quantity of caregiver-child interaction. Rather than provide a structured activity, field workers asked the caregiver to play and engage with the child as naturally as possible. Interactions were video-recorded using a tripod-supported digital camera for later frequency coding; field workers remained in the home to manage the camera and record notes as needed. The observations took place following the interview and spot-check to allow for the establishment of rapport beforehand and to minimize participants’ discomfort. We selected thirty minutes as the duration for the observations to ensure that enough time was allotted to capture participants’ typical behaviors even in cases where the child or caregiver were initially reacting to the field worker and camera.

We applied an adapted version of the Indicator of Parent-Child Interaction (IPCI) to evaluate the extent to which parents engaged with their children in ways that promote communication, learning, and positive social-emotional behaviors [55]. Caregiver behaviors of interest included four facilitating behaviors (Conveys acceptance and warmth; Uses descriptive language; Follows child’s lead; Maintains or extends child’s focus/interest), and two interrupting behaviors. The first interrupting behavior (Uses restrictions/intrusions) was part of the original IPCI and indicates negative behavioral direction; our research team added a second interrupting behavior (Uses interruptions with an explanation) to account for positive behavioral direction. In accordance with the authors’ user manual, the six behaviors were coded as “0-never occurs” to “3-often and consistently occurs” for each caregiver-child dyad [55]. The IPCI was selected due to its strong psychometric properties, close alignment with our research team’s conceptualization of positive caregiving behavior, and because the four-point rating scale would allow us to capture more variation as compared to scales with dichotomous indicators [56].

#### Key informant interviews

We conducted key informant interviews (*N*=12) with *Cuna Más* daycare instructors. Interviews were steered by a field guide that focused on instructors’ experiences working at the daycare centers and their perceptions of challenges to fostering positive home learning environments in the local communities. The data collectors audio-recorded the interviews with participant consent and documented them through field notes.

### Data management and analysis

Quantitative data from the spot-checks and structured sections of the interviews were double entered into a data management program and analyzed using Stata 13 (StataCorp LP, College Station, Texas, USA). We characterized distributions of variables by frequency or by mean and standard deviation and disaggregated by age group where relevant. We analyzed variables related to children’s engagement with play-based activities as both categorical and dichotomous variables for ease of interpretation.

For analysis of the video observations, we derived the initial codebook (with behaviors of interest, definitions, and examples) from the IPCI manual. Two members of the study team watched the first few videos simultaneously and worked collaboratively to extract examples and non-examples of each behavior and to supplement the code definitions with specific details grounded in the video data. Several codes were also added inductively at this point. The two study team members then independently coded ten (34.5%) of the 29 observations using the refined codebook and discussed and resolved discrepancies, thus finalizing the codebook. The remaining videos were divided between the two team members. For each 30-min observation, the frequencies of codes were tallied independently for ten-minute segments and then an overall rating was applied for each behavior, thus accounting for both frequency and consistency.

Extended field notes recorded during the interviews with caregivers and daycare instructors were supplemented with transcriptions from the audio recordings and independently coded by two members of the study team using ATLAS.ti software (Scientific Software Development, Berlin, Germany). Study team members developed codebooks for each set of qualitative data using a priori codes derived from the developmental niche framework and the study objectives, as well as inductive codes that emerged during data analysis. We had ongoing discussions with field workers throughout the analytic process in order to validate interpretations of the data.

### Ethical approval

The research protocol was approved by the ethics committees at the Johns Hopkins School of Public Health (Baltimore, MD) and Asociación Benéfica PRISMA (Lima, Peru). Adult participants provided written informed consent for all study components and granted permission for each child to participate in the direct observation. Participants were identified by anonymous ID numbers and data confidentially was ensured at all levels.

## Results

We triangulated relevant quantitative and qualitative findings to determine the defining features of the home learning environments. Below, we begin with an overview of participant characteristics and then present our findings related to physical and social settings, childcare practices, and caregivers’ psychology.

### Participant characteristics

A total of 30 caregiver-child dyads participated in the study, with 10 from each of the three child age groups. As displayed in Table 2, the majority of caregivers were mothers of the index child; two grandmothers and an aunt also participated. Most caregivers were between 21 and 40 years of age, had completed secondary school, and had improved water and sanitation infrastructure in their homes.

**Table 2:** Socio-demographic characteristics (*N*=30)

Characteristic	n (%)
Child age group (months)	
6–11	10 (33.3)
12–17	10 (33.3)
18–23	10 (33.3)
Caregiver’s relationship to child	
Mother	27 (90.0)
Grandmother	2 (6.7)
Aunt	1 (3.3)
Caregiver age (years)	
< 21	3 (10.0)
21–30	15 (50.0)
31–40	9 (30.0)
> 40	3 (10.0)
Caregiver educational achievement	
Primary or secondary school incomplete	7 (23.3)
Secondary school complete	23 (76.7)
Number of adults (> 18 years) in household	
2	5 (16.7)
3	5 (16.7)
4 or more	20 (66.7)
Number of children/youth (< 18 years) in household	
1	1 (3.3)
2	11 (36.7)
3	10 (33.3)
4 or more	8 (26.7)
Source of drinking water for household	
In-home piped connection	24 (80.0)
Well	4 (13.3)
Other	2 (6.7)
Sanitation facility for household	
Flush toilet	20 (66.7)
Unprotected pit latrine	10 (33.3)

### Physical and social settings for play

The homes in peri-urban *Villa El Salvador* were generally in close proximity to one another and multi-generational. In many cases, extended families lived in larger homes where young couples with one or more children each occupied a single room. Among our study population, approximately two-thirds of the households included four or more adults, and all but one household had at least two children (Table 2). As a result, children played with siblings, cousins, or other relatives that were close in age on a daily basis. As one mother of a ten-month-old boy with two siblings reflected, “*His brother is a huge help—without him, he wouldn’t have anyone to play with. The two of them are always over there, playing [gesturing to bedroom].”*

Study participants reported that these playtimes rarely took place outside, given residents’ safety concerns; several caregivers mentioned recent increases in gang activities and theft, and alluded to a lack of trust in their neighbors. Most men worked in the informal sector and were generally away from home during the day. Nevertheless, 20 (66.7%) caregivers reported that the child’s father provided some attention or care to the child every day.

Within the homes, toys and other learning materials were present to varying extents. Based on our spot-check data, the most common types of toys were those classified by the HOME-IT as “cuddly or role-playing toys” (e.g., stuffed animals, dolls, action figures); these were present in 24 (80.0%) homes and popular among all age groups (Table 3). Of these, stuffed animals were the most common, with more than one present in the majority of households. “Motor activity toys or equipment” (i.e., balls, rattles, rocking horses) and “simple hand-eye coordination toys” (i.e., rattles, marbles, and stones) were each present in 22 (73.3%) homes and were slightly more common among the youngest age group (some items were double-coded into these two categories). “Complex hand-eye coordination toys” (i.e., blocks, jigsaw puzzles) were the least common category of toys, present in 40.0% of households across all age groups. Only two (6.7%) households had any type of “puzzle” game, while 11 (36.7%) had building blocks or Lego-like toys. During the semi-structured interviews, caregivers’ reporting of children’s activities generally aligned with the presence of toys at home: while playing with balls was very popular (90.0% of children did this at least several times per week), the majority of children had never used building blocks or playsets (Table 4). Greater variability was noted with respect to playing with electronic toys (i.e., toys that produce sounds, lights, music, or words when child touches a button): while nearly two-thirds of children played with such toys often or every day, more than one-third of children had never played with them given a lack of ownership.

**Table 3:** Categories of learning materials by child age group^a^ (*N=*30)

Category	Examples of toys	6–11 mo. (***n=***10) *n*	12–17 mo. (***n***=10) *n*	18–23 mo. (***n=***10) *n*	Total (***N***=30) *n (%)*
**Cuddly toys or role-playing toys**	Stuffed animals, dolls, action figures	9	8	7	24 (80.0)
**Motor activity toys or equipment**	Bat, ball, rattle, rocking horse	9	7	6	22 (73.3)
**Simple hand-eye coordination toys**	Ball, rattle, marbles, stones	9	7	6	22 (73.3)
**Push or pull toys**	Wooden cart, box with string, shoe with string, swing	5	9	6	20 (66.7)
**Toys for literature and music**	*For infant:* rattle *For toddler*: book; cup and spoon to make sounds	8	4	7	19 (63.3)
**Learning facilitators**	Mobile, table and chair, highchair, play pen	4	7	6	17 (56.7)
**Complex hand-eye coordination toys**	Blocks, jigsaw puzzle, clay toy	4	4	4	12 (40.0)
**Movement facilitators**	Hand-made wooden walker, kiddie car, tricycle	5	4	0	9 (30.0)

^a^Based on spot-check data; categorized according to the “Learning materials” domain of HOME-IT

**Table 4:** Children’s engagement in play-based activities (*N=*30)

Activity	6–11 mo. (***n=***10) *n*	12–17 mo. (***n=***10) *n*	18–23 mo. (***n=***10) *n*	Total (***N=***30) *n (%)*
***Structured interactive activities***				
Household member reading a book to the child				
Never	5	7	3	15 (50.0)
Occasionally	0	0	0	0
Often	4	3	6	13 (43.3)
Every day	1	0	1	2 (6.7)
Household member telling stories to the child				
Never	4	8	6	18 (60.0)
Occasionally	0	0	0	0
Often	4	1	3	8 (26.7)
Every day	2	1	1	4 (13.3)
Household member singing to child				
Never	0	1	1	2 (6.7)
Occasionally	0	0	0	0
Often	3	4	4	11 (36.7)
Every day	7	5	5	17 (56.7)
***Other play-based activities***				
Using building blocks				
Never	7	7	4	18 (60.0)
Occasionally	0	0	0	0
Often	3	1	4	8 (26.7)
Every day	0	2	2	4 (13.3)
Using everyday objects found around the house as toys				
Never	2	0	0	2 (6.7)
Occasionally	1	0	0	1 (3.3)
Often	3	4	5	12 (40.0)
Every day	4	6	5	15 (50.0)
Pretending with toys				
Never	7	2	0	9 (30.0)
Occasionally	0	0	0	0
Often	2	5	4	11 (36.7)
Every day	1	3	6	10 (33.3)
Playing with balls				
Never	1	0	0	1 (3.3)
Occasionally	0	0	0	0
Often	5	5	6	16 (53.3)
Every day	4	5	4	13 (43.3)
Using electronic toys that say words, letters, or numbers when the child touches a button or image				
Never	3	5	3	11 (36.7)
Occasionally	0	1	0	1 (3.3)
Often	3	3	5	11 (36.7)
Every day	4	1	2	7 (23.3)
Watching TV programs or videos				
Never	2	0	1	3 (3.3)
Occasionally	0	0	0	0
Often	1	2	1	4 (13.3)
Every day	7	8	8	23 (76.7)

“Toys for literature and music” were present in 19 (63.3%) homes. Books for young children were recorded in nine (30.0%) homes, eight of which had only one or two books in total. During the spot-checks, field workers also noted that books for older children, which were provided by the government for public education, were present in approximately two-thirds of the homes. In terms of music, rattles were somewhat common (43.3%), yet other toys for making music, such as a plastic tambourine, were recorded in only nine (30.0%) households. In addition to the items specified on the spot-check instrument, caregivers reported that children used a number of other everyday objects as toys, including plastic soda bottles, plastic cups, clothespins, and empty containers. Fifteen (50.0%) caregivers reported that their children used household objects house as toys everyday (Table 4). At least one color-television set was present in all homes.

### Childcare practices surrounding play and communication

Observation data revealed that caregivers generally had low or inconsistent levels of interaction with their children in the context of play. As displayed in Table 5, the average score for the IPCI construct of “descriptive language” was 1.5/3, indicating that caregivers’ use of multiple-word phrases or sentences to describe activities, objects, behaviors, or feelings was relatively rare [55]. In several (13 of 29) cases, there was only one instance during the 30-min observations that the caregiver’s remarks met the definition of descriptive language, and in two cases, this did not happen at all. Caregivers overwhelmingly used single words to give commands or comment on something to a child, such as saying “*¡Patea!*” (“Kick!”) while the child played with a ball, or “*¡Gato! ¡Mira!*” (“Cat! Look!”) when pointing to a stuffed animal of a cat.

**Table 5:** Quality of caregiver-child interaction based on Indicator of Parent-Child Interaction tool (*N=*29)

IPCI construct and description	Example from observation data	IPCI score^a^ (out of a maximum of 3) *(mean, range)*
***Facilitating behaviors***		
**Conveys acceptance and warmth** Displaying warmth through verbal (i.e., making positive comments to child) and nonverbal (i.e., gentle, affectionate touch) signals	Child stacks two blocks together and caregiver responds, “Great job! Bravo!”	1.8 (0, 3)
**Uses descriptive language** Describing activities, objects, and/or child’s behaviors or feelings with multiple-word sentences	Caregiver asks child, “Where is your shoe?”; Caregiver says “Let’s go to the store”	1.5 (0, 3)
**Follows child’s lead** Noticing what interests the child and imitating, joining, turn-taking, or commenting appropriately on it	Child picks up ball and throws it to caregiver, and caregiver throws it back	1.4 (0, 3)
**Maintains/extends child’s focus** Introducing activities or materials, or using words/gestures in a novel way to engage child that demonstrates consideration to child’s interests	Child is looking at book and caregiver begins pointing out the animals on each page	0.6 (0, 2)
***Interrupting behaviors***		
**Uses restrictions/ intrusions** Making short, restrictive statements, or taking things away or controlling child’s movement unnecessarily	Child reaches for cell phone and caregiver says “No!” sharply	0.9 (0, 3)
**Uses interruptions with an explanation** Interrupting a child’s behavior while offering a verbal explanation or learning objective	Child throws a toy and caregiver says calmly, “Don’t throw that, you could hurt someone”	0.5 (0, 2)

^a^Frequency of each construct within participants’ 30-min video observations, coded based on the Indicator of Parent-Child Interaction (IPCI) tool’s scoring system (0-Never; 1-Rarely/mild; 2-Sometimes/inconsistent; 3-Often/consistently)

Accordingly, several daycare instructors commented on the low levels of verbal communication in the home setting, pointing out that it was customary for caregivers to use affectionate, diminutive terms rather than longer sentences. As one instructor reported, “*The moms don’t talk clearly to them [young children]—they don’t say words and how they’re pronounced … So the majority [of children] here in this community don’t speak much.*” During the observations, children—even those close to 24 months of age—rarely said words themselves.

With regards to playing with children, observation data revealed that caregivers at times “followed the child’s lead” by joining or imitating an activity in a non-intrusive manner, such as tossing a ball back to the child. The majority of caregivers (89.7%) provided toys or other objects for the child to play with during the observation; toys were entirely absent in only two cases. However, it was far less common for caregivers to engage the child in a way that “maintains or extends the child’s focus,” described by the IPCI as a “higher order skill” as compared to simply following the child’s lead. Caregivers rarely introduced novel, stimulating activities through words, expressions, or gestures. Rather, caregivers tended to watch or leave the room once children became interested in an activity, at times leaving them to play with older siblings or other children.

Caregivers reported that they rarely interacted with their young children in the context of storytelling and reading: 18 (60.0%) and 15 (50.0%) caregivers reported that they told stories and read to children less than once a month or not at all, respectively (data not shown). In contrast, singing to children was extremely common, as the overwhelming majority of caregivers reported doing this every day, even for the youngest children (Table 4). As one mother said of her eight-month-old infant,

*I sing to him. Everything that I learned in school, the knees and toes—'Head, Shoulders, Knees and Toes.’ I sing about the little chicks, about what the different foods are, about the maracas …*. *[Gesturing] ‘The maracas, the maracas, above, above! … The maracas, the maracas, below, below!’*

Participants enthusiastically mentioned a number of different songs that they sang to their children; the most popular songs were centered around animals, such as *La Vaca Lola* (“The Cow Lola”), *El Pato Renato* (“The Duck Renato”), and *El Caracolito* (“The Little Snail”).

Several participants also mentioned singing traditional songs in Quechua (the native indigenous language of some grandparents) to their children. Other family members, including children’s older siblings, fathers, and grandmothers, also sang often. In addition, caregivers frequently played music from the radio or from their cell phones to entertain their children. A 19-year-old mother spoke of her 23-month-old child’s enthusiasm for these activities:

*Almost every night I put some music on, and he starts to dance. He loves to dance! Like this [gesturing] with his movements. He knows how to sing … most of all he likes the songs from [TV] commercials.*

The IPCI construct of “conveys acceptance and warmth” yielded the highest average score (1.8/3). Most caregivers consistently displayed warmth through nonverbal communication such as affectionate touch and smiling at the child. Caregivers also commonly demonstrated approval through verbal feedback such as saying “*¡Bravo!*” or “*¡Eso!*” (“That’s it!”) in an excited tone, often accompanied by clapping.

Observations revealed differences in the ways that caregivers communicated to redirect children’s behavior when they did something deemed inappropriate or unsafe, referred to as “interrupting behaviors” by the IPCI. The average scores for “uses restrictions/intrusions” was 0.9/3, as compared to an average score of 0.5/3 for “uses interruptions with an explanation,” reflecting that caregivers were more likely to interrupt a child’s behavior with a short command (“*No!*” or “*Leave it alone!*”) rather than provide an explanation or learning objective. In ten cases, caregivers appeared to provide an explanation when discouraging a certain behavior; for example, in one instance when a 15-month-old boy threw a toy, her mother calmly stated, “*Don’t throw that, you could hurt someone*.”

Across all age groups, caregivers frequently put the television on or played videos on their cell phones for their children, according to data collected through both observations and interviews. Eight (27.6%) observed children watched TV or cell phone videos for at least a portion of 30-min observations; in two of these cases, this activity occupied the majority of the observation period. In cases where participants were not directly watching TV, it often remained turned on: in 13 (44.8%) participating homes, the TV was on in the background for all 30 min of the direct observation.

During the interviews, 70–80% of caregivers within each child age group reported that their children watched TV every day (Table 4). For example, when describing the previous day’s activities, one mother of a 22-month-old reported,

*He woke up around 5:30 and starts watching children’s videos on the DVD. Around 7:30, he ate breakfast with his siblings, but when they have breakfast, I always turn the TV off. Around 9, I took him to the market with me … when we returned, I started the DVD again while he ate his mid-morning fruit. Then he started to play—yesterday he was playing alone during the day, but with the TV on.*

Daycare instructors also remarked on the large role that TV played in children’s daily lives, mentioning that they served as a means of entertainment as well as distraction. One instructor suggested that technology is used as a way to “*calm or quiet their children while they [the caregivers] are doing chores at home.*”

### Caregiver perspectives on play

During the interviews, caregivers displayed mixed understandings of the overall importance of play for child development. Some caregivers discussed play as having an important role for motor skills, such as walking, kicking, and “*learning how to move*,” without referencing the less observable aspects of development such as cognitive and socio-emotional growth. However, other caregivers spoke of the importance of play for thinking, learning new things, or “*waking up the mind*.”

**Fig. 1:**
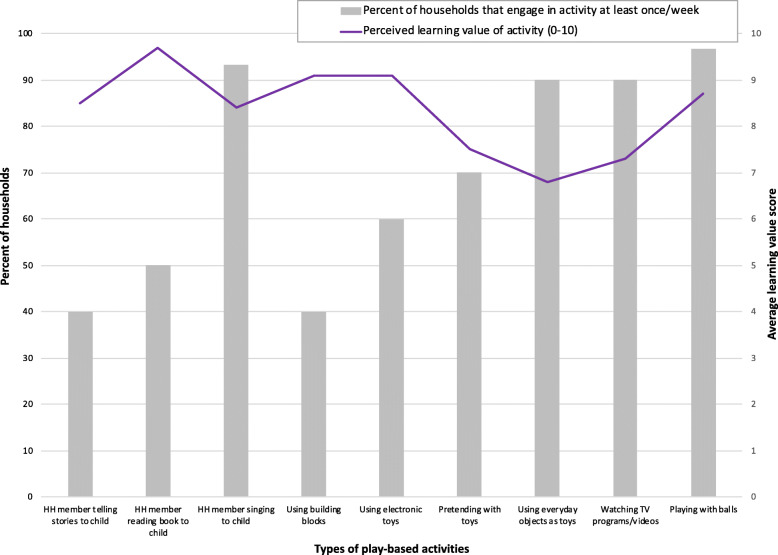
Frequency and caregivers’ perceived value of play-based activities

Caregivers’ ratings for the learning value of specific play-based activities ranged from 6.8 (for using everyday objects around the house as toys; SD 2.6) to 9.7 (for reading a book to a child; SD 0.5), out of a maximum of ten possible points. As displayed in Fig. 1, relative perceptions of learning values were not always closely aligned with how often children engaged in the activities. For example, caregivers mentioned that playing with building blocks and electronic toys had relatively high potential to assist with a child’s learning (each received an average rating of 9.1 and SD of 1.3), yet these were among the most infrequently practiced activities. In contrast, the lowest average rating was assigned to the more commonly practiced activity of using everyday objects around the house as toys.

The high rating that caregivers assigned to reading to a child suggests that the low levels of engagement with this activity were not due to a lack of perceived value. Caregivers explained that even though they knew the merits of reading, they generally did not have books for young children at home and/or did not have enough time to read, given that their days were filled with making trips to the market, domestic responsibilities such as cooking and cleaning, and accompanying other children to and from school. As one mother of three commented,

*I don’t read books to her at this time. When my [older] daughter sits down to do her homework, sometimes I show her my [older] daughter’s book … but there aren’t any books for babies in here.*

Caregivers on average assigned a moderately high learning value for the two most common activities of listening to music/singing and playing with balls (8.4 and 8.7, respectively). Interestingly, there was high variability in caregivers’ perceptions of listening to music/singing, as ratings ranged from 1 to 10; in comparison, the ratings for reading had a far smaller range of 8 to 10. Nevertheless, the participating daycare instructors unanimously spoke of the benefits of songs for learning and described their extensive use of them in the classroom. One instructor explained, “*There are a ton of songs—a ton! Songs help develop everything. For example, when you sing a song about rabbits, they are skipping, jumping, running around, looking for the carrot in the song*.”

The learning value of watching TV programs or videos received an average rating of 9.0. During the interviews, some caregivers referred to “educational” videos that they played on their smartphones from YouTube or other websites. One mother of a 23-month-old boy described how these types of programs “help to develop his mind” by differentiating colors and letters, for example.

## Discussion

In this study, we aimed to characterize the play and learning environments of young children in peri-urban Peru in order to understand potential risks to ECD and identify opportunities for intervention. Guided by the developmental niche framework, we integrated quantitative and qualitative methods to explore how physical and social settings, childcare practices, and caregiver perceptions structure children’s early experiences.

Findings shed light on several aspects of children’s home environments that may not optimally support early learning and that may provide opportunities for intervention development. First, caregivers in general displayed low levels of responsive interactions in the context of play. Although most children had access to toys in their physical settings, it was rare for caregivers to engage as play partners for extended periods of time or to introduce novel activities. Our direct observations demonstrated that in many cases children played with siblings and cousins if they were present, while caregivers watched or, at times, left the room. While these types of social and free play are important, the limited opportunities for “guided play”—in which a caregiver supports the learning experience during child-led play—and scaffolding learning may adversely affect ECD [57–59]. Improving caregivers’ skills for guided play and heightening caregivers’ awareness of their facilitative role in children’s development are important intervention goals in this setting.

Second, caregivers’ limited verbal interactions with their young children suggest that the developmental niche is not particularly supportive of language formation. Our use of the IPCI tool, which focuses on caregivers’ use of “descriptive language”—rather than just the number of words spoken to a child—demonstrated the paucity of rich and varied caregiver speech among our study population [55]. This reflects findings from other low-income settings in Latin America and sub-Saharan Africa where child-directed speech is not customary and, in contrast to Western societies, may even be stigmatized [60–63]. For example, Shneidman and Goldin-Meadow (2012) demonstrated that among Mayan communities, most of young children’s language input consists of overheard words, given that adults do not treat them as “conversational partners” and that they mainly spend time with other children rather than adults [60]. Among our study population, these trends, along with relatively low rates of reading and storytelling to children, warrant intervention prioritization given the importance of child-directed speech for vocabulary growth and cognitive development [64–67].

Opportunities for caregiver-child interactions appeared to be constrained by the regular use of TV and video programs in the home, as displayed by both our interview and observation data. Substantial evidence has demonstrated the negative developmental effects of TV-watching as well as background TV for young children, in part because caregivers are less attentive and engaged when the TV is on [68–71]. In samples of U.S. children, frequent TV usage has been associated with less developed oral language skills at 24 months of age [72]. Furthermore, although some caregivers referred to infant-directed shows as “educational,” the effectiveness of programming marketed as learning tools is not supported by the scientific literature [73, 74]. The high rates of TV exposure that we documented may be attributed to aspects of the “larger ecology,” including the lack of safety in peri-urban communities, which prevents children from playing freely outside, as well as the increasing accessibility of TVs and smartphones. According to Super and Harkness’ framework, the developmental niche is an open system and, thus, changes in the broader culture and economy may exert pressure for change on certain elements of the developmental niche [33].

Importantly, our findings demonstrate the central role that music and song play in the daily lives of our study participants and suggest opportunities for leveraging songs to enrich children’s learning environments in future interventions. Music appeared to be a fundamental and joyous part of daily life, and children more frequently listened to singing and music than any other activity. Given that a handful of songs and nursery rhymes were widely known, and that an array of family members participated in singing, there is great potential to draw on these traditions to more explicitly encourage communication and positive interactions. In intervention studies conducted in developed countries, musical activities emphasizing touch, gesturing, and vocalizations have demonstrated considerable improvements in infants’ social and language skills [75, 76]. In our study setting, caregivers could be encouraged to incorporate new gestures, expressions, and dancing while singing favorite children’s songs, just as the *Cuna Más* daycare instructors reported doing. Future caregiver-directed interventions should explore the potential of leveraging songs and sing-a-longs to improve communication and positive interactions in similar study settings.

In addition, our data suggest several ways in which households’ socio-economic realities should be considered in the design of a future home-based ECD intervention. First, interventions should emphasize and enhance the use of everyday objects around the house as toys. Although this behavior was relatively common among our study participants, its average perceived learning value was lower than any other activity. In contrast, the use of battery-powered electronic toys received the second-highest rating for perceived learning value. The advertising and packaging of electronic toys often tout their educational benefits, yet such claims are not based on scientific evidence [77–79]. In fact, the use of electronic toys may adversely affect cognitive and language development by limiting opportunities for interpersonal interactions. Sosa and colleagues (2016), for example, found that caregiver-child dyads (10–16 months) who were playing with electronic toys displayed decreased quantity and quality of caregiver language input and fewer child vocalizations as compared to those playing with traditional toys (i.e., puzzles, blocks) or board books [78]. In our study setting, a future play-based ECD intervention should address the misperception that costly, electronic toys are better learning tools, and should convey to caregivers that it is not necessary to spend money to create a developmentally supportive learning environment. Furthermore, intervention efforts should demonstrate how caregivers may enhance the learning value of everyday objects, such as creating building blocks out of recycled cartons or fashioning a rattle from an empty soda bottle and dry beans. This is particularly important given the strong evidence from low-resource settings that the variety of play materials in the home predicts motor and language development in young children [80].

Furthermore, future interventions should encourage communication in the context of caregivers’ daily commitments. Among our study participants, domestic responsibilities and time constraints led to perceptions that it was not feasible to engage with children in productive ways, such as through reading or dedicated play sessions. However, opportunities for interaction occur throughout the day. Intervention efforts targeting caregiver-child interaction should encourage caregivers to speak to their children in full sentences and identify play opportunities while doing chores in the home, going to the market, or taking part in other daily activities. The need for behavioral direction when a child acts in an unacceptable way presents further opportunities for child-directed speech. In contrast to using strict, single-word restrictions (referred to as “negative behavioral direction”), caregivers should be encouraged to provide a learning objective (“positive behavioral direction”) in these situations.

The language environment may also be enriched through the engagement of older siblings and other household members. Future home-based interventions should advise caregivers to model descriptive language for other household members and to encourage their school-aged children to talk, play, and interact with their younger siblings, even before that sibling can speak. In addition, individuals delivering such interventions should aim to engage with as many household members as possible, given that they all contribute to the home learning environment and that there are many demands on mothers’ time. Incorporating the option of conducting weekend or evening visits would likely advance these goals.

### Limitations

The study could have been limited by the following. First, self-reported behaviors may be subject to social desirability bias, which could have affected how caregivers discussed their perceptions or behaviors during the semi-structured interviews. Likewise, reactivity was a potential limitation related to the direct observations, such that participants may have altered their behaviors in the presence of field workers. However, these biases were likely minimized by the fact that data collectors underwent extensive training which emphasized engaging with study participants in a nonjudgmental manner and conveying that the information collected would be used to better understand community patterns, rather than to evaluate individual families. During direct observations, the child and caregiver stopped noticing and/or reacting to the camera after the first few minutes. In addition, the overall low levels of caregiver-child interactions observed were triangulated with interview data, and do not support concerns over reactivity.

Second, the IPCI was an imperfect tool for assessing the quality of caregiver-child interaction. As with many observations tools, the IPCI was developed with samples of caregiver-child dyads from the U.S. and thus may face challenges when applied cross-culturally [81]. In our study context, the IPCI did not capture all relevant features of young children’s interactive learning environments that we observed in practice, such as the role of siblings or cousins as play partners, or contextual aspects such as background TV noise. Fortunately, our video-recorded observations allowed us to retrospectively supplement the IPCI tool with additional variables; this represents a major advantage of using recordings rather than only applying the tool during an in-person, live observation. Ultimately, this underscores the importance of adapting tools to reflect cultural and contextual considerations, and of using multiple methods to achieve a comprehensive understanding of the home learning environment.

Finally, our qualitatively informed sample size and the cross-sectional nature of data collection suggest that our quantitative findings should not be generalized across contexts. However, they proved sufficient for achieving our formative research goals. The strengths of our study design, including the triangulation of multiple methods and the study of two types of caregivers, ensure the validity of our findings. The immersion of several co-authors in the field site and the practice of regularly discussing emerging findings with local field workers further support the study’s credibility. In addition, although our participants came from a single, peri-urban district, this study area may be comparable to other peri-urban communities in Peru or other Latin American countries, especially with regards to cultural traditions such as music and song, as well as the growing prevalence of household possessions such as TVs and mobile phones. Thus, our findings may inform future efforts to assess and intervene on home learning environments in other similar settings.

## Conclusions

In this study, we have demonstrated that the application of the developmental niche framework and the integration of quantitative and qualitative methods may provide a comprehensive understanding of children’s early learning environment, and guide intervention decision-making. Formative research is essential to developing an ECD intervention that addresses local needs, leverages local strengths, and is acceptable to the target community. Our findings indicate that there is great potential for a home-based intervention focusing on singing, play-based learning, and home-made toys to improve ECD in peri-urban Lima, Peru. One possible mechanism for delivering such a service is the government’s *Cuna Más* program, which currently provides home visiting services exclusively in the country’s rural regions and therefore has limited capacity to influence caregiver practices within peri-urban households. Future research should explore the feasibility, acceptability, and ultimately the effectiveness of such an intervention model for improving young children’s learning potential and future achievements in this setting.

## Supplementary Information

**Additional file 1.**

## Data Availability

The quantitative datasets used and analyzed during the current study are available from the corresponding author on reasonable request. The qualitative data sets that support the findings of this study are not publicly available because we did not ask participants to consent to raw data sharing outside of the research team. Public sharing of the data could compromise research participant consent.

## Abbreviations

ECD: Early child development
LMIC: Low- and middle-income country
UNICEF: United Nations Children’s Fund
WHO: World Health Organization
