# Supplementary material for: Assessing the play and learning environments of children under two years in peri-urban Lima, Peru: a formative research study
Source: BMC Public Health. 2021 Jan 9;21:108. doi: 10.1186/s12889-020-10119-3 (PMC7796591; doi:10.1186/s12889-020-10119-3)
Supplement: Supplementary file 1 — Additional file 1. [file 12889_2020_10119_MOESM1_ESM.docx]

**Supplement 1: Participant Interview Guide**

Participant ID: ___ ___ ___ Field worker ID: ___ ___ ___

Date: ___ ___ / ___ ___ / ___ ___ Sector: _________________________

Start time: ___ ___ : ___ ___ am/pm Neighborhood: ___________________

End time: ___ ___ : ___ ___ am/pm Recorder: 1 2

**Module A. Socio-demographic information**

*READ: To begin, I am going to ask a few questions about you and your child.*

|  | **Question** | **Options** | **Response** |
| --- | --- | --- | --- |
| A_1 | What is the index child’s date of birth? | Date of birth: ___ ___ / ___ ___ / ___ ___ | |
| A_2 | How old (in months) is the index chidl?  *(should be between 6 months – 2 years)* | Age (months) | ____ ____ |
| ***The following questions refer to the caregiver:*** | | | |
| A_3 | What is your relationship to this child? | 01 = Mother  02 = Father  03 = Grandmother  04 = Grandfather  05 = Aunt  06 = Uncle  07 = Neighbor  08 = Other: __________________ | ____ ____ |
| A_4 | How old are you? | Age (years) | ____ ____ |
| A_5 | What’s the highest grade of school that you have completed? | 00 = Primary incomplete  01 = Primary complete  02 = Secondary complete  03 = University/technical school | ____ ____ |
| ***The following questions refer to the home where the child is cared for:*** | | | |
| A_6 | How many adults ( > 18) live in the household? | Number of adults | ____ ____ |
| A_7 | How many children (< 18 years) live in the household? | Number of children | ____ ____ |
| A_8 | How many of these children are < 2 years? | Number of children | ____ ____ |
| A_9 | What is the status of the property? | 00 = Titled property  01 = No land title (squatting)  02 = Renting  03 = Other: _________________ | ____ ____ |
| A_10 | What is the source of water? | 00 = In-home piped connection  01 = Outside piped connection  02 = Well  03 = Water truck  04 = Purchase water  05 = From neighbor  06 = Other: _________________ | ____ ____ |
| A_11 | What type of toilet? | 00 = Flush toilet  01 = Pit latrine (unprotected)  02 = Protected pit latrine  03 = Other: _________________ | ____ ____ |

**Module B. Early Learning Environment**

*INSTRUCTIONS: Ask these questions to the primary caregiver, substituting the name of the target child where specified.*

|  | **Question** | **Options** | **Response** |
| --- | --- | --- | --- |
| B_1 | How many times per week is [NAME] taken out of the house? | 00 = Less than once/week  01 = Once/week  02 = 2 times/week  03 = 3 times/week  04 = 4 times/week  05 = More than 4 times/week | ____ ____ |
| B_2 | Do you ever take [NAME] to the market? | 00 = No  01 = Yes | ____ ____ |
| B_2a | *If the answer to 2 is “Yes*”…  How often do you take [NAME] to the market? | 00 = Never  01 = About once/month  02 = More than once/month, but less than once/week  03 = Once/week  04 = More than once/week | ____ ____ |
| B_3 | Is [NAME] regularly cared for by someone other than the parents? | 00 = No  01 = Yes | ____ ____ |
| B_3a | *If the answer to 3 is “Yes*”…  How many people regularly provide care for [NAME]? | Number of people | ____ ____ |
| B_3b | *If the answer to 3 is “Yes*”…  Who regularly cares for [NAME]? | 01 = Older sibling  02 = Grandmother  03 = Aunt  04 = Grandfather  05 = Uncle  05 = Family friend  06 = Other (specify):_______ | ____ ____  ____ ____  ____ ____  ____ ____ |
| B_4 | Does [NAME]’s father provide some care every day? | 00 = No  01 = Yes | ____ ____ |
| B_5 | Do you read stories to [NAME]? | 00 = No  01 = Yes | ____ ____ |
| B_5a | *If the answer to 5 is “Yes*”…  How often do you read stories to [NAME]? | 00 = Once/month or less  01 = A few times/month  02 = Once or twice/week  03 = Three or more times/week | ____ ____ |
| B_6 | Does [NAME] eat meals with his/her mother and father? | 00 = No  01 = Yes | ____ ____ |
| B_6a | *If the answer to 6 is “Yes*”…  How often does [NAME] eat meals with mother and father? | 00 = Less than once/week  01 = A few times/week  02 = Once/day  03 = More than once/day | ____ ____ |
| B_7 | Does [NAME] regularly visit relatives or receive visits from relatives? | 00 = No  01 = Yes | ____ ____ |
| B_7a | *If the answer to 7 is “Yes*”…  How often does this occur? | 00 = Never  01 = About once/month  02 = More than once/month, but less than once/week  03 = Once/week  04 = More than once/week | ____ ____ |
| B_8 | How many books of his/her own does [NAME] have? | 00 = None  or  Number of books | ____ ____ |

**Module C. Play and learning activities**

**Module C, Part 1: Open-ended questions**

*READ: Now I’m going to ask you a few open questions about how you and your child spend your time. Please share as much or as little detail as you would like.*

C_1. Thinking about yesterday, can you walk me through what [NAME] did all day, during the morning, afternoon, and evening?

- About how much time did [NAME] spend sleeping, eating, playing, watching T.V., etc.?
- Who was [NAME] with at different times of the day?

C_2. Would you consider yesterday to be a “normal” day? If not, how is a “normal” day different from what you described about yesterday?

C_3. When you spend time with [NAME], do you play with him/her?

- In what ways do you play?
- Do you use toys with him/her?

C_4. In your opinion, is playing important for helping your child to learn and develop? Why or why not?

C_5. When you spend time with [NAME], do you read books?

- What kinds of books?
- What are the names of the books? Can I see them?

C_6. When you spend time with [NAME], do you tell made-sup stories?

- What kinds of stories?

C_7. Do you ever sing or hum when you are spending time with [NAME]?

- What kinds of songs or melodies do you sing? Can you sing them?
- Does anyone else in the household sing or hum when spending time with the child?

**Module C, Part 2: Frequency of activities**

*READ: For this part, we’re interested on how often your child does certain things. For each of the activities that I’m going to read, please tell me whether your child does it rarely or never; about once per month; a few times per month; about once per week; a few times per week; or every day.*

|  | **Question** | **Response**  **(circle one)** |
| --- | --- | --- |
| C_8 | Using building blocks or play sets | 00 = Rarely or never  01 = About once per month  02 = A few times per month  03 = About once per week  04 = A few times per week  05 = Every day |
| C_9 | Using everyday objects found around the house as toys (e.g. pots/pans, plastic containers, etc.) | 00 = Rarely or never  01 = About once per month  02 = A few times per month  03 = About once per week  04 = A few times per week  05 = Every day |
| C_10 | Pretending with toys (e.g. a doll, toy cars, action figures) | 00 = Rarely or never  01 = About once per month  02 = A few times per month  03 = About once per week  04 = A few times per week  05 = Every day |
| C_11 | Having a book read to them | 00 = Rarely or never  01 = About once per month  02 = A few times per month  03 = About once per week  04 = A few times per week  05 = Every day |
| C_12 | Play with balls | 00 = Rarely or never  01 = About once per month  02 = A few times per month  03 = About once per week  04 = A few times per week  05 = Every day |
| C_13 | Listening to music or a family member singing or humming | 00 = Rarely or never  01 = About once per month  02 = A few times per month  03 = About once per week  04 = A few times per week  05 = Every day |
| C_14 | Having a story told to them | 00 = Rarely or never  01 = About once per month  02 = A few times per month  03 = About once per week  04 = A few times per week  05 = Every day |
| C_15 | Using electronic products that say words, letters, or numbers when child/baby touches a button, word, or picture | 00 = Rarely or never  01 = About once per month  02 = A few times per month  03 = About once per week  04 = A few times per week  05 = Every day |
| C_16 | Sitting quietly watching TV programs or videos | 00 = Rarely or never  01 = About once per month  02 = A few times per month  03 = About once per week  04 = A few times per week  05 = Every day |
| C_17 | Watching TV programs or videos and singing, dancing, or interacting with the show | 00 = Rarely or never  01 = About once per month  02 = A few times per month  03 = About once per week  04 = A few times per week  05 = Every day |

**Module C, Part 3: Value of activities for learning**

*READ: Now we would like you to rate each of the activities mentioned in terms of its ability to set a foundation for learning. Use a rating scale of 1-10 for each activity, where 1 means “This activity definitely does NOT set a foundation for learning” and 10 means “This activity definitely sets a foundation for learning.”*

|  | **Question** | **Response**  **(circle one)** |
| --- | --- | --- |
| C_18 | Using building blocks or play sets | 1 2 3 4 5 6 7 8 9 10 |
| C_19 | Using everyday objects found around the house as toys (e.g. pots/pans, plastic containers, etc.) | 1 2 3 4 5 6 7 8 9 10 |
| C_20 | Pretending with toys (e.g. a doll, toy cars, action figures) | 1 2 3 4 5 6 7 8 9 10 |
| C_21 | Having a book read to them | 1 2 3 4 5 6 7 8 9 10 |
| C_22 | Playing with balls | 1 2 3 4 5 6 7 8 9 10 |
| C_23 | Listening to music or a family member singing or humming | 1 2 3 4 5 6 7 8 9 10 |
| C_24 | Having a story told to them | 1 2 3 4 5 6 7 8 9 10 |
| C_25 | Using electronic products that say words, letters, or numbers when child/baby touches a button, word, or picture | 1 2 3 4 5 6 7 8 9 10 |
| C_26 | Sitting quietly watching TV programs or videos | 1 2 3 4 5 6 7 8 9 10 |
| C_27 | Watching TV programs or videos and singing, dancing, or interacting with the show | 1 2 3 4 5 6 7 8 9 10 |

**Module C, Part 4: Perceptions of activities**

*READ: Now I’m going to ask a few more questions about your opinions of different activities.*

C_28. What is your child’s favorite activity?

C_29. Were any activities mentioned that you wish your child engaged in more often? Which one(s)?

C_30. What kinds of TV programs or video does [NAME] watch? During what part or parts of the day?

C_31. Is there anything else that you’d like to add about the topics discussed today that you haven’t mentioned yet?
